# Large-Pore Channels at the Maternal–Fetal Interface: Progress and Open Research Avenues

**DOI:** 10.3390/biology15181571

**Published:** 2026-09-08

**Authors:** José L. Vega, Antonia Moral, Camila Gutiérrez, Juan C. Sáez

**Affiliations:** 1 Departamento de Fisiología, Facultad de Ciencias Biológicas, Universidad de Concepción, Concepción 4070386, Chile; amoral2022@udec.cl; 2 Departamento de Ciencias Biológicas y Químicas, Facultad de Ciencias, Universidad San Sebastián, Concepción 4080871, Chile; camila.gutierrez@uss.cl; 3 Instituto de Neurociencia, Centro Interdisciplinario de Neurociencia de Valparaíso (CINV), Universidad de Valparaíso, Valparaíso 2340000, Chile; juancarlos.saez@uv.cl

**Keywords:** connexin, pannexin, CALHM, LRRC8, trophoblast, syncytialization, preeclampsia, maternal–fetal interface

## Abstract

The maternal–fetal interface functions as an integrated physiological unit whose homeostatic balance determines pregnancy success. Large-pore channels, composed of connexins (Cxs), pannexins (Panxs), calcium homeostasis modulators (CALHMs) and leucine-rich repeat-containing 8 (LRRC8) proteins, mediate direct intercellular communication, autocrine and paracrine release of ATP and other signaling molecules, and scaffold-based signal integration across this interface. In this review, we synthesize current knowledge on large-pore channel expression and their physiological and pathophysiological roles at the maternal–fetal interface, applying an explicit evidence-classification framework to distinguish established functions from emerging findings. We first map the large-pore channel repertoire of each cellular compartment—syncytiotrophoblast, cytotrophoblast, extravillous trophoblast, villous endothelium and decidual stroma—revealing that each compartment expresses a distinct combination of family members, with varying levels of evidence and gestational stage-dependent dynamics. We then analyze the three principal modes of large-pore channel operation in placental physiology: gap-junctional communication driving syncytialization, channel-mediated ATP release enabling paracrine purinergic signaling, and channel-independent scaffolding functions that integrate mechanical and biochemical signals. Next, we examine how each channel family becomes dysregulated in placental-related diseases. We conclude by outlining a targeted research roadmap with clear priorities: the most urgent need is protein-level validation of CALHM and LRRC8 expression in primary trophoblasts, followed by elucidation of gating mechanisms and testing for crosstalk among channel families. By providing both conceptual synthesis and practical guidance, this review aims to accelerate mechanistic understanding and therapeutic development targeting large-pore channels for pregnancy complications that currently lack mechanism-based treatments.

## Simple Summary

Healthy pregnancies depend on constant communication between the mother’s body and the developing baby through the placenta. Special protein gates, known as large-pore channels, allow placental cells to share essential signals, exchange nutrients, and coordinate growth. When these cellular channels fail to work properly, it can lead to serious pregnancy complications that currently lack effective, targeted treatments. In this review, we summarized what is known about these protein gates throughout different stages of pregnancy and across various cell types in the placenta. We identified three main ways these channels work: linking neighboring cells together, releasing signaling molecules into their surroundings, and acting as structural hubs inside cells. We also examined how breakdowns in these channels contribute to placental diseases. Finally, we outlined priority research steps to guide future studies. By providing a clear blueprint of how cells communicate in the placenta, this work will help researchers design new, targeted therapies to prevent and treat pregnancy complications, ultimately protecting the health of both mothers and their babies.

## 1. Introduction

The human placenta is a transient organ whose function determines lifelong health trajectories for both mother and offspring [1,2]. Far from being a passive diffusion barrier, the maternal–fetal interface operates as a dynamic, multicellular unit whose integrated activity supports gas exchange, nutrient transport, hormone production and immunological tolerance throughout gestation [1,2]. This functional unit comprises several specialized cell types operating in tight coordination. The syncytiotrophoblast (STB), a continuous, multinucleated epithelial layer devoid of lateral borders, forms the primary exchange surface bathed directly by maternal blood [1,2]. Beneath it lie mononucleated cytotrophoblasts (CTB) that proliferate and fuse to maintain and expand the syncytium. At anchoring villi, CTB differentiates into extravillous trophoblasts (EVT) that invade the decidua and remodel maternal spiral arteries, ensuring adequate blood supply to the intervillous space [1,2]. The fetal side comprises villous capillary endothelium, while the maternal contribution includes decidual stromal cells and a diverse array of immune cells that modulate trophoblast behavior. This architectural complexity demands sophisticated communication mechanisms to coordinate cellular activities across compartments and over gestational time [1,2].

In vertebrates, four families of proteins that form large-pore channels—Cxs, Panxs, CALHMs, and LRRC8—have emerged as key mediators of intercellular, autocrine, and paracrine communication in diverse tissues [3,4,5]. Despite distinct evolutionary origins and divergent structural architectures, all four families share the defining functional capacity to conduct ions and small organic molecules up to approximately 1 kDa [3,4,5]. However, important differences in permeability, selectivity and gating exist (Table 1). The Cx family oligomerizes into hexameric hemichannels (connexons) that can either dock with connexons from adjacent cells to form gap-junction channels, establishing direct cytoplasmic continuity, or operate unopposed at the plasma membrane to release autocrine and paracrine signals [6,7,8]. The Panx family typically forms heptameric plasma membrane channels that release ATP upon activation by membrane depolarization, mechanical stretch or caspase cleavage during apoptosis [9,10,11,12], and some authors suggest they form gap junction channels, which have received less attention [13]. The CALHM family assembles into large oligomeric channels (octamers to dodecamers) gated by voltage and extracellular calcium ions, with established roles in gustatory neurotransmission, immunological synapse, and neuronal excitability [14,15,16,17,18,19,20,21]. The LRRC8 family constitutes volume-regulated anion channels (VRACs) that mediate regulatory volume decrease through efflux of Cl^−^ and organic osmolytes upon hypotonic swelling or shrinkage caused by osmotic challenges during cell growth, division and migration [22,23,24,25,26,27,28,29,30].

**Figure 1:**
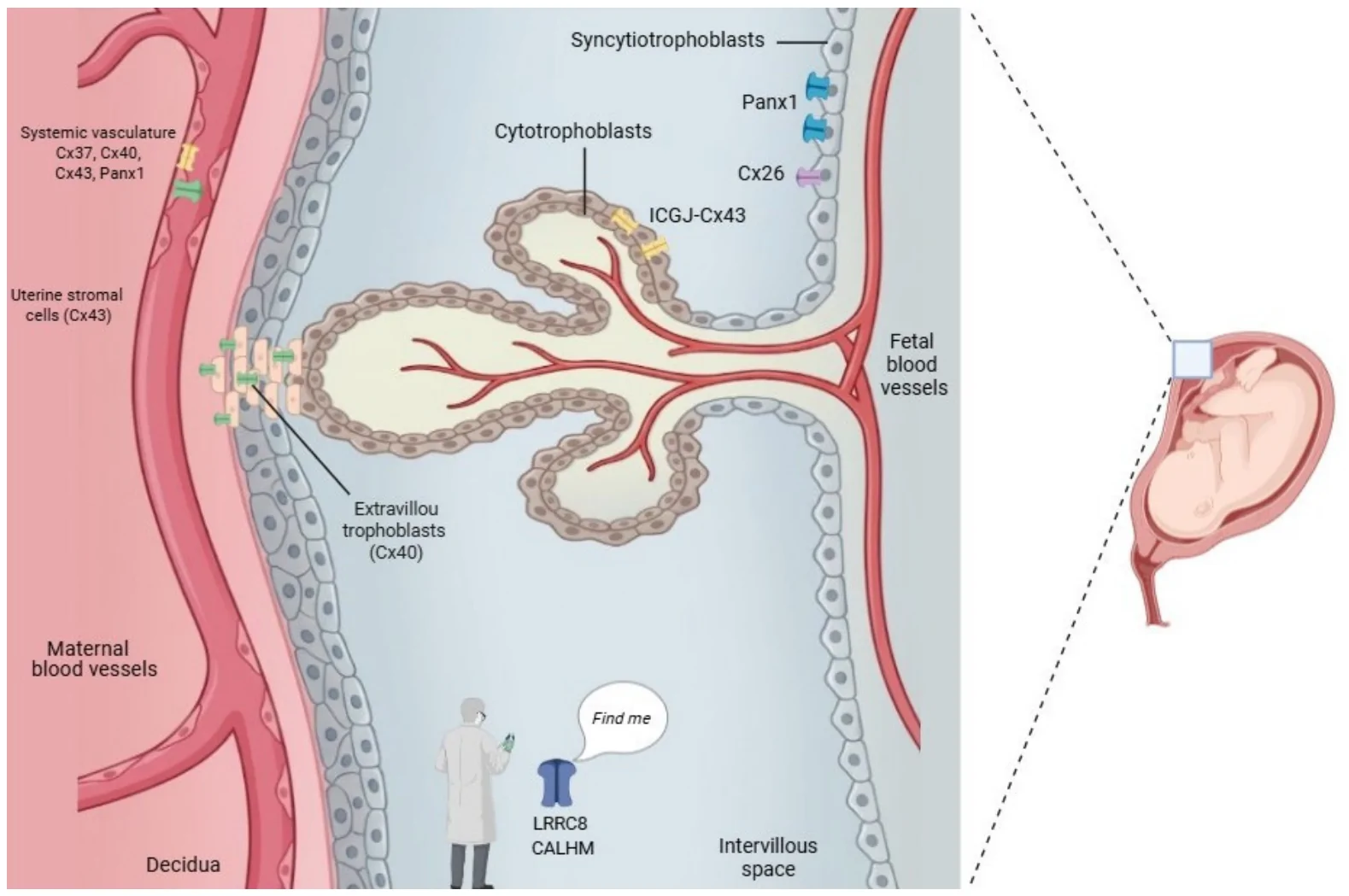
Large-pore channel localization at the maternal–fetal interface. Schematic illustration showing the cellular distribution and relative expression of major large-pore channel families—Cxs, Panxs, CALHMs, and LRRC8/VRACs—across maternal decidual cells and distinct placental trophoblast subpopulations. Note that the cellular expression and functional roles of CALHM and LRRC8/VRAC channels remain largely uncharacterized and warrant further investigation. Created in BioRender. Jose L. Vega. (2026). https://app.biorender.com/illustrations/6a99ce78d41c5b5af36640c1.

The human placenta expresses members of all four families (Figure 1). Transcriptomic data confirm the presence of Cx26, Cx31, Cx40, Cx43 [31,32,33], Panx1 [34], CALHM2, CALHM4, CALHM6 [15], and LRRC8 subunits [35] (Table 2). Whether these channels operate independently, serving redundant or specialized functions, or whether they interact with each other, remains an open question. Two excellent reviews comprehensively described the background of Cxs and Panxs in female reproductive organs, providing a thorough update up to 2015 [32,33] (Table S1). The present review advances this knowledge by extending the update to 2026 and, importantly, incorporating two additional protein families: LRRC8 and CALHM. To guide readers in critically interpreting the literature, we evaluate the evidence surrounding large-pore channels in the human placenta using an explicit, three-tiered framework. Level I (Established) encompasses findings supported by multiple independent studies and complementary approaches in primary human cells. Level II (Emerging) comprises reproducible evidence requiring further functional validation in physiological contexts, typically derived from immortalized cell lines, animal models, or trophoblast organoids. Level III (Hypothetical) represents hypothesis-generating observations based on limited expression data, indirect inferences from other tissues, or early-stage technologies. Critical Gap highlights fundamental unanswered questions where evidence is absent, contradictory, or insufficient to formulate even a tentative hypothesis—these are priority areas where basic knowledge is lacking and that impede translational progress. By clearly delineating well-validated roles from speculative hypotheses and identifying critical unknowns, this framework provides a transparent assessment of the current state of the field (Table S2). Our goal is to provide a conceptual blueprint that guides future investigation toward mechanistic understanding and, ultimately, therapeutic intervention.

## 2. The Large-Pore Channel Repertoire at the Placental Maternal–Fetal Interface

Understanding how large-pore channels contribute to placental function first requires knowing which channels are expressed, in which cells, and with what confidence. This section maps the channel repertoire of each cellular compartment at the maternal–fetal interface, applying the evidence-classification framework to guide critical interpretation.

### 2.1. Syncytiotrophoblast

The STB is the primary effector layer of the placenta, a continuous multinucleated epithelial layer that transports nutrients, secretes hormones, and forms the primary barrier between maternal blood and fetal circulation. Its unusual structure poses unique challenges for intercellular communication and places special demands on membrane channel systems [36]. **Cxs:** The STB expresses Cx43 as its predominant Cx, with protein localized at residual CTB–STB interfaces and within the syncytial cytoplasm [31,37,38]. Cx43 is lost from the fully differentiated syncytial membrane, suggesting that it functions primarily during fusion rather than in maintaining the differentiated state. Lower levels of Cx26 have been detected in STB, particularly at terms, where it may contribute to the membrane permeability to small molecules [32] [II]. Cx46 has also been detected at low levels in trophoblast lineages, although its functional relevance remains unclear [39] [II]. **Panxs:** Panx1 is abundantly expressed in STB and exhibits dynamic regulation [34] [II]. Since Panx2 and Panx3 have highly restricted tissue distributions, their expression has not been found in the human placenta at functional or detectable levels so far. A key methodological insight emerged from studies of placental explants: the characteristic uptake of propidium iodide by STB during early ex vivo culture, long misinterpreted as cell death, results from Panx1 upregulation rather than membrane disruption [34] [II]. Panx1 protein increases markedly within 3 h of culture, independent of oxygen tension, and pharmacological blockades with probenecid prevent dye uptake without affecting viability [34]. However, probenecid also blocks organic anion transporters; therefore, genetic approaches or selective and potent Panx1 channels are required to definitively assign Panx1 function in placental explants. These findings have critical practical implications: studies using explant systems must account for Panx1-mediated permeability as a confounding variable. **CALHMs:** Expression analysis revealed abundant mRNA for CALHM2, CALHM4 and CALHM6 in term placental tissue, with levels exceeding those of many well-characterized transport proteins [15] [II]. All three paralogues are upregulated during in vitro trophoblast differentiation, in primary trophoblast cells isolated from healthy term placentae, with CALHM4 showing the most dramatic induction during syncytialization [15] [II]. These observations currently originate from a single group and report only transcript presence without protein-level confirmation. Transcript analysis confirmed the above findings, while functional characterization in HEK293 cells revealed that activation of these paralogs differs from the voltage- and calcium-gated channel CALHM1, and cryo-EM structures showed that CALHM4 forms decameric and undecameric assemblies with a large cylindrical pore and CALHM6 exhibits a conical pore that narrows at the intracellular side [15]. However, these functional and structural experiments were performed in heterologous systems, not in primary human trophoblasts. The upregulation of CALHM transcripts during trophoblast differentiation strongly suggests a relevant physiological role, but no study has directly demonstrated CALHM channel function (electrophysiology, ATP release, or ion flux) in primary human trophoblast cells. Consequently, validating CALHM expression, subcellular distribution and function in primary syncytiotrophoblasts remains an imperative next step [Critical Gap]. **LRRC8:** Direct evidence for LRRC8 expression in STB is currently absent. The case rests on incidental findings from studies not designed to investigate placental VRAC function. Kubota et al. (2004) noted placental expression while characterizing LRRC8 in B-cell development [35] [III]. That study does not provide cell-type resolution or protein-level confirmation. To date, no study has performed functional assays (electrophysiology, ion flux measurements, regulatory volume decrease, or ATP release) on LRRC8/VRAC channels in human trophoblast cells. Protein-level, cell-type-specific expression in primary trophoblasts has not been confirmed. Thus, the presence of functional VRACs in STB remains a hypothetical [Critical Gap].

### 2.2. Cytotrophoblasts and Extravillous Trophoblasts

The mononucleated CTB population serves as the progenitor pool for both the overlying syncytium and the invasive extravillous lineage [40]. **Cxs.** First-trimester CTBs express a repertoire of Cxs. Cx40 and Cx45 predominate in anchoring cell columns, where CTBs maintain a proliferative phenotype [41] [II]. Functional studies suggest that Cx40-mediated gap-junctional communication maintains the proliferative state. Conversely, inhibition of Cx40 triggers a transition to an invasive EVT phenotype [38] [II]; however, these studies were performed in JEG-3 human choriocarcinoma cells and require validation in primary CTBs. The possible interaction between hypoxia and Cx40 has been studied in JEG-3 cells: low Cx40 levels prioritize cell migration, whereas high Cx40 levels maintain cell proliferation [42] [II]. Cx31 is also expressed in CTBs, and its dysregulation has been linked to trophoblast dysfunction, although mechanistic studies are limited [32] [II]. **Panxs:** The mRNA of three pannexin genes (*PANX1*, *PANX2*, *PANX3*) have been detected in cultured explants using qRT-PCR methodology [34] [II]. However, their regulation in the progenitor population remains unexplored. **CALHMs and LRRC8:** Expression patterns of CALHM and LRRC8 family members in CTBs and EVTs have not been systematically investigated. This represents a critical knowledge gap that warrants targeted transcriptomic and proteomic studies with cell-type resolution [Critical Gap].

### 2.3. Villous Endothelium

Villous endothelial cells express Cx37, Cx40 and Cx43 [32] [II]. Cx40 is particularly abundant and shows dynamic regulation: acute chorioamnionitis is associated with Cx40 upregulation in placental vasculature, although whether this reflects a protective or pathological response remains unresolved [42] [II]. In the systemic vasculature, Cx37, Cx40 and Cx43 form gap junction channels that coordinate vasomotor responses and maintain endothelial barrier function, and they also form hemichannels that mediate purinergic signaling [43]. While Cx40 is required for conducted vasodilation and blood flow regulation [44], Cx37 regulates endothelial monolayer integrity and leukocyte adhesion [45]. Cx43, the most ubiquitously expressed Cx in endothelium, has been implicated in angiogenesis, shear-stress sensing and inflammatory activation [43,44,46]. Insights from murine knockout models reinforce these specialized yet overlapping vascular roles. While single ablation of Cx37 or Cx40 yields viable animals, their simultaneous deletion—as well as the knockout of Cx45—results in embryonic or perinatal lethality due to defective vasculogenesis and mural cell recruitment. Furthermore, targeting specific isoforms frequently triggers secondary alterations; for example, Cx40 deficiency destabilizes endothelial Cx37 and disrupts Cx43 subcellular distribution, demonstrating that vascular homeostasis depends on a tightly coordinated Cx network [45]. To date, there is no direct evidence confirming the presence of Panx1 in the villous endothelium of the human placenta. While Panx1 expression has been clearly demonstrated in other endothelial types, including human umbilical vein endothelial cells [47], aortic endothelial cells [48], and microvascular endothelium [49] of various organs, its specific localization in placental villous endothelial cells remains unexplored [Critical Gap]. Recent studies have demonstrated that the mechanosensitive channel Piezo1 is expressed and functional in human fetoplacental endothelium, where it detects shear stress induced by blood flow [50]. Piezo1-dependent ATP release in endothelial cells is mediated, in part, by Panx1 channels [51]. Notably, activation of Piezo1 facilitates Ca^2+^ influx, followed by activation of CaMKII, which phosphorylates Panx1 at S394, increasing the activity of Panx1 channels [52]. However, whether Panx1 is expressed in the villous endothelium and participates in this mechanotransduction pathway remains unknown [Critical Gap].

### 2.4. Decidual Stroma

Decidual stromal cells express high levels of Cx43, which is essential for decidualization. Ovarian steroids precisely regulate endometrial Cx expression: estrogens upregulate Cx26 and Cx43, whereas progesterone suppresses their expression as the uterus transitions to a receptive state [53,54,55] [II]. In human endometrial stromal cells, Cx43 upregulation precedes the expression of classical decidualization markers, and its knockdown impairs differentiation [56] [I]. Stromal-specific GJA1 deletion in mice prevents decidualization, impairs angiogenesis, and causes pregnancy loss [57,58] [I]; however, caution is needed when extrapolating to human decidualization owing to differences regarding of trophoblast invasion and the temporal regulation of Cxs.

### 2.5. Immune Cells

Immune cells, particularly natural killer (NK) cells and macrophages, express Cxs and pannexins in other tissues [59,60,61,62]. However, no studies to date have specifically investigated the expression, regulation, or functional role of Panx1 or Cx43 in the immune cells that reside at the maternal–fetal interface [Critical Gap]. Given emerging roles for purinergic signaling in maternal–fetal immune tolerance [63,64], this represents a priority area for investigation. In conventional NK cells, Cx43 forms gap junctions that facilitate intercellular communication with target cells and with other immune effectors, modulating cytotoxicity and cytokine production [59,65]. In macrophages, Cx43 and Panx1 channels are central to inflammasome activation and the release of pro-inflammatory cytokines, including IL-1β and IL-18 [62,66,67,68]. Uterine NK cells and decidual macrophages are phenotypically distinct from their peripheral counterparts, exhibiting a more regulatory and less cytotoxic profile [69,70]. Therefore, there is a compelling need for the identification and characterization of Cx43 and Panx1 expression in purified uterine NK cells and macrophages from first trimester and term decidua, as well as functional studies using selective channel inhibitors or genetic models and co-culture systems that recapitulate the maternal–fetal interface.

## 3. Physiological Roles of Large-Pore Channels in the Placenta

**Figure 2:**
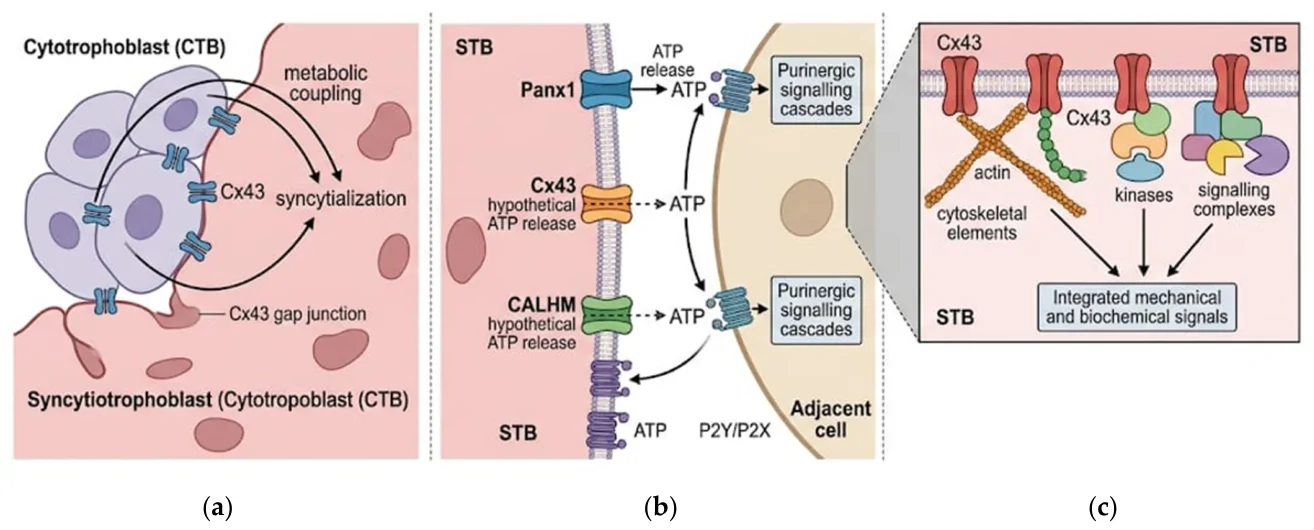
Proposed modes of large-pore channel operation in the syncytiotrophoblast. Three proposed mechanisms through which large-pore channels may contribute to STB function include the following: (**a**) Gap-junctional communication between CTB and/or STB mediated by connexins (Cx45, Cx40, Cx31, Cx43, Cx46, Cx26), putatively coordinating syncytialization and metabolic coupling. (**b**) Hemichannel-mediated ATP release via Panx1, Cx43, or CALHM channels (established, solid arrow; hypothetical, dashed arrow), which may activate P2Y/P2X receptors on STB and adjacent cells to initiate purinergic signaling cascades. (**c**) Putative channel-independent scaffolding functions of Cx43, potentially interacting with cytoskeletal elements (ZO-1, tubulin, actin) and signaling complexes to integrate mechanical and biochemical signals.

Large-pore channels may contribute to placental cell–cell communication through at least three distinct mechanisms: direct intercellular coupling via gap junctions, autocrine and paracrine signaling via hemichannel-mediated release, and channel-independent scaffold functions (Figure 2).

### 3.1. Direct Communication: Gap Junctions and Syncytialization

Gap junctions formed by Cxs enable direct cytoplasmic continuity between adjacent cells, allowing passage of ions, second messengers (e.g., cAMP, cDAPR, IP_3_, and Ca^2+^) and small metabolites up to approximately 1 kDa [71]. In the placenta, gap-junctional communication serves a fundamental role in syncytialization, the continuous fusion of mononucleated CTBs into the multinucleated STB, which is the defining dynamic process of placental development. The fusion of CTBs into the STB specifically requires Cx43 [31,37] [I]. siRNA-mediated silencing of GJA1 significantly impairs syncytialization in vitro, reducing multinucleated cell formation and human chorionic gonadotropin secretion [37] [I]. Mechanistically, fusion requires PKA-mediated phosphorylation of Cx43 at Ser369 and Ser373, induced by the cAMP pathway [72,73] [II]. These phosphorylation events regulate not only gap-junction assembly but also interactions with scaffold proteins, positioning Cx43 as a signaling hub that coordinates both electrical coupling and cytoskeletal remodeling required for membrane mergers. Of note, ultrastructural evidence for gap junctions at the CTB–STB interface is limited; most studies localize Cx43 to the CTB plasma membrane and to cytoplasmic pools, rather than to the nascent fusion site [31]. Beyond Cx43, other large-pore channel families may also participate in syncytialization. The dynamic upregulation of CALHM4 mRNA during syncytialization raises the possibility that CALHM channels contribute to this process, perhaps by releasing ATP or other signals that modulate fusion [15] [III]. Whether Panx1 or LRRC8/VRACs play any role in syncytialization is entirely unknown [Critical Gap]. The established role of Cx43 positions it as a master regulator; however, Cx43-dependent syncytialization is not unique to the placenta. In skeletal muscle, myoblast fusion during myotube formation requires Cx43, and its downregulation impairs the formation of multinucleated myofibers [74,75]. Cx43 has also been found to participate in the formation of bone osteoclast-like foreign body giant cells, which are formed in response to the implantation of biomaterials such as nanoparticulate hydroxyapatite [76]. These observations across distinct syncytial systems suggest that Cx43 plays a conserved and non-redundant role in cell–cell fusion events, yet the molecular mechanism—whether Cx43 acts as hemichannel, gap junction channel, and/or scaffolding protein—remains unresolved.

### 3.2. Autocrine and Paracrine Communication and ATP Release

Unopposed Cx hemichannels (connexons), Panx channels, VRAC, and CALHM channels release ATP into the extracellular space, activating purinergic receptors on adjacent cells and initiating autocrine or paracrine signaling cascades [77,78,79,80]. It is important to note that not all large-pore channels contribute equally to ATP release: Panx1 channels and CALHMs are established ATP-release pathways in other systems [78,79]. Also, Panx3 hemichannels allow the release of ATP in other systems [81], while Cx26, Cx31.1, Cx32, Cx36, Cx43, Cx45, and Cx46 hemichannels serve as pathways for ATP release with distinct selectivity—the N-terminal domain together with the N-terminus transmembrane 2 region have been proposed as key molecular determinants [82], and LRRC8/VRACs release predominantly glutamate and aspartate with modest ATP permeability [80].

Panx1 is expressed in the STB and exhibits dynamic regulation in placental explants, where its upregulation drives propidium iodide uptake—an observation that has important methodological implications for viability staining [34] [II]. However, while Panx1 is a well-established ATP-release channel in other cell types [77,78], direct experimental evidence for Panx1-mediated ATP release specifically in primary human trophoblasts is currently lacking. The available data demonstrate Panx1 expression and channel activity (dye uptake), but ATP release has not been directly measured, nor have the downstream purinergic receptors been identified in this cell type. Thus, the functional role of Panx1 in placental purinergic signaling remains to be established [Critical Gap].

Moreover, no study has directly measured ATP release via Cx43 hemichannels in primary human trophoblasts. However, in other cell types, Cx43 hemichannels are well established to release ATP and other signaling molecules in response to depolarization [6], low extracellular Ca^2+^ [6], metabolic inhibition [6], mechanical stimulation [6], nitrosylation induced by nitric oxide [83,84,85], and proinflammatory conditions [86]. Whether Cx43 hemichannels contribute to purinergic signaling in trophoblasts under physiological or pathological conditions has not been directly tested. To date, the available evidence in placenta is limited to Cx43 expression and regulation, with no functional studies demonstrating hemichannel-mediated ATP release in this cell type. Therefore, this remains an open question [Critical Gap].

CALHM channels are established ATP-release pathways in other systems: CALHM1 and CALHM3 mediate ATP release from taste bud cells during gustatory signaling [79,87], and CALHM2 has been implicated in astrocytic ATP release [88]. The abundant mRNA levels of CALHM2, CALHM4 and CALHM6 in trophoblasts strongly suggests they subserve ATP-release functions [15] [III], but to our knowledge, direct experimental evidence in placental cells is completely absent [Critical Gap].

A critical missing link in this pathway is whether trophoblasts express P2Y and P2X receptors. While P2 receptors have been described in the placenta, systematic cell-type-specific mapping is lacking. This is essential because ATP release does not equate to purinergic signaling without receptor expression, ectonucleotidases, and downstream effectors. This represents a priority area for future investigation.

### 3.3. Decidual Invasion and Vascular Remodeling

EVT invasion into the decidua and remodeling of maternal spiral arteries are essential for establishing adequate placental perfusion [89,90,91]. These processes require proliferation of EVT progenitors in anchoring cell columns, transition to an invasive phenotype, interactions with decidual cells and immune cells, and endovascular invasion with replacement of the endothelial lining [89,90,91]. Cx40 emerges as a key regulator of the proliferation–invasion switch. In first-trimester anchoring columns, high Cx40 expression correlates with proliferative EVTs, whereas Cx40 downregulation accompanies the transition to an invasive phenotype [41] [II]. In JEG-3 cells, Cx40 levels modulate hypoxia responses: low Cx40 levels are associated with cell migration, whereas high Cx40 levels occur during cell proliferation [92] [II]. These findings suggest that Cx40-mediated gap-junctional communication maintains EVTs in a proliferative state and that its downregulation permits invasion. However, these studies were conducted in immortalized human choriocarcinoma cells, and validation in primary EVTs or organoid models is required before this can be considered established. Decidual Cx43 is essential for decidualization, and its dysregulation may impair the decidual response to invading EVTs [56,57,58,93] [I]. Adiponectin exerts anti-differentiative effects by downregulating Cx43 via ADIPOR1/2, limiting trophoblast invasion through altered MMP/TIMP balance, suggesting a mechanistic link between maternal metabolic status and placental invasion [93] [II]. The potential roles of the CALHM and LRRC8 families in invasion have not been investigated [Critical Gap].

### 3.4. Channel-Independent Scaffolding Functions

Beyond their ion and molecule-conducting functions, large-pore channel proteins, particularly Cxs, serve as scaffolding platforms that organize signaling complexes and anchor the cytoskeleton [94,95]. These channel-independent functions are well established in other systems but remain underexplored in the placenta [8]. The C-terminal domain of Cx43 interacts with zonula occludens-1 (ZO-1), microtubules, and actin-binding proteins, positioning Cx43 as a nexus for integrating mechanical and biochemical signals in trophoblastic cells [72,73,96] [II]. Specifically, PKA-mediated phosphorylation of Cx43 at Ser373 disrupts its interaction with ZO-1, allowing connexons to mobilize to the plasma membrane and form functional hemichannels or gap junctions [72,96] [II]. During syncytialization, these interactions may coordinate the extensive cytoskeletal remodeling required for membrane fusion. Pidoux et al. (2010) demonstrated that ZO-1 is involved in trophoblastic cell differentiation in human placenta, providing direct evidence for scaffold functions in this tissue [96] [II]. The observation that Cx43 knockdown impairs syncytialization could reflect loss of channel function, scaffolding function or both, a distinction not yet addressed experimentally [37] [I]. Panx, CALHM and LRRC8 proteins have not been reported to have channel-independent functions, but systematic investigation is lacking.

## 4. The Large-Pore Channel Families in Placenta-Related Diseases

The previous sections described the expression and physiological roles of each large-pore channel family. This section evaluates available evidence for their dysregulation in placenta-related diseases, focusing on each family independently while noting that co-occurrence of changes does not imply interaction. A comprehensive overview of their reported alterations, functional mechanisms, and remaining literature gaps is summarized in Table 3.

### 4.1. Preeclampsia

Preeclampsia, a multisystem disorder characterized by new-onset hypertension and proteinuria after 20 weeks of gestation, affects 2–8% of pregnancies globally and remains a leading cause of maternal and perinatal mortality [97]. Its pathophysiology involves two overlapping stages: poor placental perfusion followed by a maternal systemic inflammatory response [97]. Preeclamptic placentas exhibit an aberrant Cx signature defined by marked Cx43 upregulation alongside a concomitant reduction in Cx46 [39] [II]. Consistently, trophoblast Cxs exhibit distinct oxygen-dependent sensitivities in vitro: while hypoxia sustains elevated levels of phosphorylated and total Cx43, it drives the progressive downregulation of Cx46 [39] [II]. Panx1 is significantly upregulated in the preeclamptic placenta [98] [II]. Emerging evidence links Panx1 to ferroptosis, an iron-dependent regulated cell death pathway, in preeclampsia. Clinical data show Panx1 upregulation in preeclamptic placenta [98] [II]. Mechanistic studies in trophoblast lines suggest that Panx1 promotes ferroptosis through an ATF3-dependent pathway that suppresses glutathione peroxidase 4 (GPX4), impairing neutralization of lipid peroxides [98,99] [II]. An upstream regulatory mechanism has been identified: downregulation of miR-224-5p leads to Panx1 overexpression, elevated reactive oxygen species (ROS) and accelerated ferroptosis, impairing trophoblast proliferation and invasion [99] [II]. Restoration of miR-224-5p in a mouse preeclampsia model attenuates hypertension and improves live birth rates [99] [II]. However, these initial clinical studies were limited by small sample sizes (*n* < 30), and current mouse models utilizing miR-224-5p antagomirs do not fully mimic human preeclampsia pathology. Consequently, while the miR-224-5p–Panx1–GPX4 axis represents a compelling therapeutic target, this concept is still preliminary and necessitates validation in more translational animal models [99] [II]. Expression of CALHMs in preeclamptic placenta has not been reported, and given their trophoblast-enriched expression and upregulation during differentiation, investigation is urgently needed. LRRC8/VRAC expression in preeclampsia remains entirely unknown [Critical Gap].

### 4.2. Recurrent Pregnancy Loss

Recurrent pregnancy loss affects 1–2% of couples and often involves failed establishment of the maternal–fetal interface [100]. First-trimester placental tissues from women with recurrent pregnancy loss show reduced Cx43 and VEGF expression compared with controls [101] [II]; however, this study comprised a small cohort and could not establish causality. Similarly, a subsequent study of 15 women with unexplained recurrent early pregnancy loss—at least three consecutive first-trimester miscarriages—reported reduced Cx43 gap junction gene expression in placental tissue [102] [II]. Together, these observational findings suggest a possible link between Cx43 downregulation and recurrent pregnancy loss, but larger prospective studies are needed to determine whether this association is causal. Panx1 expression has not been systematically investigated in recurrent pregnancy loss, and expressions of the CALHM and LRRC8 channel families in this condition remain completely unexplored [Critical Gap].

### 4.3. Fetal Growth Restriction

Fetal growth restriction affects approximately 10% of pregnancies and results from inadequate placental nutrient supply [103,104]. For instance, a dominant-negative GJA1 mutation (Cx43ᴳ^60^ˢ) causes fetal growth restriction in mice despite enhancing angiogenesis within implantation chambers [105,106] [I]. Given the substantial differences in trophoblast invasion and decidualization between mice and humans, these observations highlight that Cx effects depend on channel activity, scaffold functions, and cellular context. In fetal growth restriction, the role of the Panx, CALHM and LRRC8 channel families remains unexplored [Critical Gap].

### 4.4. Preterm Birth and Chorioamnionitis

Preterm birth, delivery before 37 weeks of gestation, complicates 5–18% of pregnancies and is a leading cause of neonatal morbidity and mortality [107]. Chorioamnionitis, an inflammation of the fetal membranes, is a major contributor to early preterm birth [108]. The strongest link to preterm birth involves Cx43 in amniotic membrane integrity. Mechanical strain and trauma upregulate Cx43 in amniotic membrane defects, activating myofibroblasts and amplifying inflammatory signaling [109,110,111] [II]. Cx43 antisense oligonucleotides applied to ex vivo membrane defect models reduce PGE2 production and enhance collagen and elastin deposition, restoring barrier integrity [110] [II]—a promising therapeutic approach awaiting in vivo validation. Separately, acute chorioamnionitis is associated with Cx40 upregulation in placental vasculature, although whether this is causal or consequential to inflammation remains unresolved [42] [II]. In murine models of *Porphyromonas gingivalis* infection, a periodontal pathogen that induces systemic inflammation rather than ascending infection, chronic inflammation triggers premature myometrial Cx43 upregulation and preterm delivery; however, the relevance to human spontaneous preterm birth requires investigation [112] [II]. While direct evidence in the inflamed amniotic membrane is currently lacking, Panx1 might play a role in this context, considering its well-documented participation in inflammasome pathways [III]. The roles of CALHM and LRRC8 in preterm birth remain to be studied [Critical Gap].

### 4.5. Genetic Variants in Large-Pore Channel Genes and Their Association with Pregnancy Complications

The strongest evidence concerns GJA1 (Cx43): although no common polymorphisms have been unequivocally linked to pregnancy complications in humans, reduced decidual Cx43 expression has been consistently reported in women with recurrent early pregnancy loss [102] and in endometriosis-related subfertility [113]; moreover, mice harboring a dominant-negative GJA1 G60S mutation exhibit severe disruption of decidual angiogenesis, leading to dysmorphic placentation, fetal growth restriction and reduced litter viability [106], while pharmacological inhibition of Cx43-mediated gap junction intercellular communication with mefloquine has been associated with increased risk of early pregnancy loss and stillbirth [114], and preeclampsia has been linked to phosphorylation-induced closure of Cx43 channels [115]. For GJA4 (CX37), the C1019T polymorphism has been investigated as a potential risk factor for spontaneous abortion, although results remain inconclusive [116]. For Panx1, several heterozygous and homozygous missense variants have been identified as monogenic causes of oocyte death and female infertility [117,118,119], relevant to the broader spectrum of reproductive disorders. By contrast, no pathogenic variants or disease-associated polymorphisms have yet been described for the CALHM or LRRC8 families (Table 4).

## 5. Conclusions

Several limitations must inform interpretation of both the existing literature and future studies. We highlight these here to provide practical guidance for researchers entering the field. Antibody Specificity: Many large-pore channel antibodies lack rigorous validation. This is particularly acute for CALHM and LRRC8 families, where commercial antibodies are often used without knockout verification or peptide competition. We recommend that all studies report validation data including knockout verification, peptide competition, and orthogonal methods (e.g., mRNA–protein correlation). For CALHM proteins, no validated antibodies have been described to date as reacting reliably in placental tissue, representing a critical barrier. Model System Limitations: Immortalized cell lines. Studies using JEG-3, BeWo or HTR-8/SVneo cells require validation in primary cells or organoids, as these lines exhibit substantial epigenetic and functional drift. For example, the Cx40–hypoxia–invasion link has been established primarily in JEG-3 cells; whether this holds in primary EVTs is unknown. Human–mouse differences: Mouse and human placentae differ fundamentally in hemochorial architecture, trophoblast invasion depth and Cx isoform repertoire. Findings from rodent models should be considered hypothesis-generating rather than directly translatable, especially for decidualization and EVT invasion. Explant culture artifacts: Panx1 is dramatically upregulated within hours of ex vivo culture [34], confounding viability assessments and potentially altering channel activity. Researchers should use fresh tissue or optimized culture conditions (e.g., low oxygen, supplemented media) and include Panx1 inhibitors as controls. Trophoblast organoids: These emerging models offer a promising bridge between cell lines and in vivo studies, recapitulating trophoblast differentiation and polarity. However, organoids lack vascular and immune components, limiting their use for studying maternal–fetal interactions. Causal Evidence: Most clinical data are correlative, and establishing causality requires genetic or pharmacological perturbation in physiologically relevant models. The field should prioritize functional experiments (e.g., CRISPR/Cas) over descriptive expression studies. Pharmacological Selectivity: A major challenge in dissecting the physiological contributions of large-pore channels stems from the promiscuity of classical pharmacological inhibitors at standard working concentrations. For instance, carbenoxolone exerts broad inhibitory effects on Panx1 [12,120] and Cx hemichannels [121]; it concurrently blocks voltage-gated Ca^2+^ channels [122] and enzymes such as 11β-hydroxysteroid dehydrogenase [123]. Similarly, probenecid exerts broad inhibitory effects on organic anion transporters (OATs) [124] while simultaneously functioning as a positive allosteric modulator of TRPV2 [125]. To date, sequence-targeted mimetic peptides (e.g., Gap26, Gap27, ^10^panx1) represent specific pharmacological tools available [126]. Also, D4, a small organic molecule, is a potent and selective blocker of some Cx hemichannels and does not affect Panx1 hemichannels or CALHM1 channels [127]. Consequently, pharmacological blockade alone provides presumptive evidence, underscoring that rigorous functional attribution necessitates genetic validation using inducible and cell specific approach.

In this review, we have synthesized the evidence for large-pore channel function at the human maternal–fetal interface, applying an explicit evidence-level framework throughout. Cxs, particularly Cx43, are established master regulators of syncytialization, decidualization and placental morphogenesis, and their dysregulation in preeclampsia, recurrent pregnancy loss and preterm birth positions them as validated therapeutic targets. Panx1 has established roles in STB membrane permeabilization and emerging roles in ferroptotic cell death in preeclampsia via a miR-224-5p-mediated mechanism; probenecid, a Panx1 inhibitor with an acceptable safety profile, offers a readily available tool for preclinical testing, though its specificity must be confirmed using genetic approaches. CALHM channels are abundantly and dynamically expressed in trophoblasts, but their activation mechanism and functional roles remain completely undefined, making resolution of this gating paradox the essential prerequisite for mechanistic progress. LRRC8 expression rests on indirect, incidental data, and the case for function relies entirely on physiological analogy; protein-level, cell-type-specific expression profiling in primary trophoblasts is therefore the immediate priority.

A fundamental open question is whether circulating gestational hormones—particularly estrogen, progesterone, and hCG—regulate the expression, trafficking, gating, or biophysical properties of large-pore channels at the maternal–fetal interface. Although indirect evidence from non-placental tissues suggests that subsets of these channels are hormone-sensitive [73,128,129,130,131], direct mechanistic investigations in primary human trophoblasts are currently lacking. Elucidating this endocrine axis could uncover novel pathways linking hormonal status to placental permeability.

At present, there is no evidence indicating that these four channel families function as a coordinated network; instead, they are best conceptualized as distinct molecular entities characterized by unique expression profiles, gating mechanisms, and physiological roles. Whether functional interplay occurs through shared permeants or mutual cross-regulation remains an unresolved and critical question. Addressing this complexity requires advanced methodological approaches. Recent breakthroughs in single-cell and spatial transcriptomics have fundamentally transformed our understanding of trophoblast heterogeneity and differentiation. Notably, the spatial multiomics map of the early maternal–fetal interface has revealed unprecedented cellular diversity and transcriptional dynamics [132]. Complementing these in vivo mapping efforts, three-dimensional trophoblast organoids derived from primary or stem cells have emerged as robust platforms for modeling placental development, function, and pathogen susceptibility [133,134]. Together, these models provide a physiologically relevant yet experimentally tractable system to dissect the cell-type-specific expression and functional roles of Cxs, Panxs, CALHMs, and LRRC8s, effectively bridging the gap between transcriptomic data and functional validation. Despite these technological advances, several fundamental knowledge gaps persist. Systematically translating these insights into definitive functional mechanisms demands a concerted experimental strategy, which we outline below as a prioritized research roadmap (Table 5).

## Figures and Tables

**Table 1:** Structural and functional properties of large-pore channel families in vertebrates.

	Connexins	Pannexins	LRRC8/VRAC	CALHM
Stoichiometry	Hexameric assemble.	Heptameric assemble.	Heptameric assemble.	Heptameric, octameric, decameric, and undecameric.
Ion selectivity	Moderate: Cx37/40 anion-preferring; Cx43/26 cation-preferring.	Weakly selective, permeable to large cations and anions.	Anion-selective (Cl^−^ >> cations).	Slightly Ca^2+^-permeable, also conducts ATP.
Gating mechanisms	Voltage, Ca^2+^, pH, phosphorylation; lipid-mediated pH gating (Cx46/50); lipid dependence of channel conformations (Cx32).	Voltage, Ca^2+^, ATP, mechanical stress; C-terminal activating domain promotes opening; distinct hexamer vs. heptamer properties; unique ion selection motifs.	Cell swelling, membrane stretch, low ionic strength.	Extracellular Ca^2+^, voltage, depolarization; cytoplasmic plug and allosteric modulation.
Pharmacological blockers	Carbenoxolone, mefloquine, Gap26/27.	Carbenoxolone, probenecid, spironolactone (Panx1).	DCPIB, NS3728, carbenoxolone (low affinity).	Ruthenium red, Gd^3+^, mefloquine.
Permeability	ATP, glutamate, NAD^+^, IP_3_, glucose (up to ~1 kDa).	ATP, UTP, Ca^2+^, dyes up to ~900 Da.	Cl^−^, organic osmolytes (taurine, myo-inositol), glutamate, ATP.	ATP, Ca^2+^
Structural resolution (cryo-EM)	Cx46/50; Cx32; Cx43; Cx36; Cx26.	Panx1; Panx2; Panx3.	LRRC8A-containing complexes.	CALHM1, CALHM 2, CALHM 4, CALHM 6.

**Table 2:** Large-pore channel expression across the maternal–fetal interface.

Cell Compartment	Cxs (Specie)	Panxs (Specie)	CALHMs (Specie)	LRRC8 *
Syncytiotrophoblast	Cx43 (human) Cx26 (human)	Panx1 (human)	CALHM2 (human) CALHM4 (human) CALHM6 (human)	Unexplored
Cytotrophoblast and Extravillous trophoblast	Cx40 (human) Cx45 (human) Cx31 (mouse)	Panx1 (human)	Unexplored	Unexplored
Villous endothelium	Cx37 (human) Cx40 (human) Cx43 (human)	Unexplored	Unexplored	Unexplored
Decidual stroma	Cx43 (human)	Unexplored	Unexplored	Unexplored
Immune cells	Unexplored	Unexplored	Unexplored	Unexplored

* LRRC8 transcripts reported in bulk placentas but no cell type or protein resolution [35].

**Table 3:** Involvement of large-pore channel families across placenta-related pathologies.

Pathology	Cxs	Panxs	CALHMs	LRRC8s
Preeclampsia	Cx43: Upregulated in preeclamptic placenta [39]. Cx46: Downregulated [39].	Panx1: Significantly upregulated [97,98]. Panx1: Promotes trophoblast ferroptosis via the miR-224-5p/Panx1/ATF3/GPX4 axis [99].	Unexplored	Unexplored
Recurrent Pregnancy Loss	Cx43: Downregulated in first trimester [100,101,102].	Unexplored	Unexplored	Unexplored
Fetal Growth Restriction	Cx43: A dominant-negative mutation (G60S) causes fetal growth restriction in mice despite enhancing angiogenesis [103,104,105,106].	Unexplored	Unexplored	Unexplored
Preterm Birth and chorioamnionitis	Cx43: Upregulated in amniotic membrane defects [107,108,109,110,111]. Cx40: Upregulated in placental vasculature during acute chorioamnionitis [42].	Unexplored	Unexplored	Unexplored

**Table 4:** Genetic variants and polymorphisms in large-pore channel genes associated with pregnancy complications.

Gene	Variant/Polymorphism/Mechanism	Associated Complication	Reference
GJA1 (CX43)	Reduced expression (possible regulatory variants)	Recurrent early pregnancy loss	[102]
GJA1 (CX43)	Reduced expression (possible regulatory variants)	Endometriosis-related subfertility	[113]
GJA1 (CX43)	G60S dominant-negative mutation (mouse model)	Abnormal placentation, IUGR, embryonic loss	[106]
GJA1 (CX43)	Pharmacological inhibition (mefloquine)	Early pregnancy loss, stillbirth	[114]
GJA1 (CX43)	Phosphorylation-induced channel closure	Preeclampsia (hypertension)	[115]
GJA4 (CX37)	C1019T polymorphism	Spontaneous abortion	[116]
PANX1	Missense variants (heterozygous, homozygous)	Oocyte death/female infertility	[117,118,119]
CALHM/LRRC8	None described	None described	—

**Table 5:** Research roadmap.

Priority	Action	Expected Outcome
Immediate Priorities (0–2 years)	Generate protein level, cell type resolved expression maps of all large pore channel families in first trimester, term and pathological placentae using multiplex immunohistochemistry and mass spectrometry.	Confirmation of LRRC8 protein expression; development or validation of CALHM antibodies.
	Establish trophoblast organoid biobanks from multiple donors with protocols for differentiation towards STB and EVT lineages.	Standardized model system for functional studies.
	Map purinergic receptor (P2Y, P2X) expression in trophoblast subtypes and decidual immune cells.	Essential missing link for ATP signaling studies.
Mechanistic Elucidation (2–4 years)	Resolve the CALHM gating paradox: screen potential activators in primary trophoblasts and organoids; test heteromeric assembly requirements; identify interacting partners by proximity labeling.	First functional characterization of CALHMs in placenta.
	Characterize VRAC (LRRC8) function in primary trophoblasts: demonstrate swelling activated chloride currents; determine LRRC8 subunit composition; assess regulatory volume decrease capacity in LRRC8A knockdown organoids.	Definitive proof of functional VRACs (LRRC8) in trophoblasts.
	Test Panx1 function using genetic approaches (CRISPR, siRNAs) rather than probenecid, in primary cells and organoids.	Unambiguous assignment of Panx1 specific roles.
Cross talk and Integration (3–5 years)	Multi gene CRISPR perturbations in trophoblast organoids (single, double and triple knockouts of GJA1, PANX1, CALHM4, LRRC8A).	Discovery of additive or synergistic effects; evidence for functional integration.

## Data Availability

No new data were created or analyzed in this study. Data sharing is not applicable to this article.

## Supplementary Materials

The following supporting information can be downloaded at: https://www.mdpi.com/article/10.3390/biology15181571/s1, Table S1: A decade (2015–2025) of progress in placental connexin research [135,136,137,138,139]; Table S2: Evidence classification justification for key claims.

## Abbreviations

The following abbreviations are used in this manuscript:

ADIPOR1/2	Adiponectin Receptor 1 and 2
Ca^2+^	Calcium Ion
CALHM	Calcium Homeostasis Modulator
CaMKII	Ca^2+^/Calmodulin-Dependent Protein Kinase II
cAMP	Cyclic Adenosine Monophosphate
cDAPR	Cyclic ADP-Ribose
CTB	Cytotrophoblasts
Cx	Connexin
EVT	Extravillous Trophoblasts
GJA1	Gap Junction Protein Alpha 1
GPX4	Glutathione Peroxidase 4
HIF-1*α*	Hypoxia-Inducible Factor 1 alpha
IP_3_	Inositol 1,4,5-Trisphosphate
LRRC8	Leucine-Rich Repeat-Containing 8
MMP	Matrix Metalloproteinases
NK	Natural Killer
OATs	Organic anion transporters
Panx	Pannexin
ROS	Reactive Oxygen Species
STB	Syncytiotrophoblast
VEGF	Vascular Endothelial Growth Factor
VRAC	Volume-Regulated Anion Channel
TIMP	Tissue Inhibitors of Metalloproteinases
ZO-1	Zonula Occludens-1

## References

[1] Kramer A.C., Jansson T., Bale T.L., Powell T.L. (2023). Maternal-fetal cross-talk via the placenta: Influence on offspring development and metabolism. Development.

[2] Jansson T., Powell T.L. (2007). Role of the placenta in fetal programming: Underlying mechanisms and potential interventional approaches. Clin. Sci..

[3] Contreras J.E. (2025). Current advances in large-pore channels: From structure-function to physiology and disease. J. Physiol..

[4] Gaete P.S., Kumar D., Fernández C.I., Valdéz Capuccino J.M., Bhatt A., Jiang W., Lin Y.C., Liu Y., Harris A.L., Luo Y.L. (2024). Large-pore connexin hemichannels function like molecule transporters independent of ion conduction. Proc. Natl. Acad. Sci. USA.

[5] Syrjanen J., Michalski K., Kawate T., Furukawa H. (2021). On the molecular nature of large-pore channels. J. Mol. Biol..

[6] Garcia I.E., Contreras J.E. (2026). Decoding Connexin Hemichannels: Structure, Function, and Regulatory Mechanisms. Annu. Rev. Physiol..

[7] Lee H.J., Cha H.J., Jeong H., Lee S.N., Lee C.W., Kim M., Yoo J., Woo J.S. (2023). Conformational changes in the human Cx43/GJA1 gap junction channel visualized using cryo-EM. Nat. Commun..

[8] Lucaciu S.A., Leighton S.E., Hauser A., Yee R., Laird D.W. (2023). Diversity in connexin biology. J. Biol. Chem..

[9] Deng Z., He Z., Maksaev G., Bitter R.M., Rau M., Fitzpatrick J.A.J., Yuan P. (2020). Cryo-EM structures of the ATP release channel pannexin 1. Nat. Struct. Mol. Biol..

[10] He Z., Zhao Y., Rau M.J., Fitzpatrick J.A.J., Sah R., Hu H., Yuan P. (2023). Structural and functional analysis of human pannexin 2 channel. Nat. Commun..

[11] Hussain N., Apotikar A., Pidathala S., Mukherjee S., Burada A.P., Sikdar S.K., Vinothkumar K.R., Penmatsa A. (2024). Cryo-EM structures of pannexin 1 and 3 reveal differences among pannexin isoforms. Nat. Commun..

[12] Ruan Z., Orozco I.J., Du J., Lu W. (2020). Structures of human pannexin 1 reveal ion pathways and mechanism of gating. Nature.

[13] Palacios-Prado N., Soto P.A., López X., Choi E.J., Marquéz-Miranda V., Rojas M., Duarte Y., Lee J., González-Nilo F.D., Sáez J.C. (2022). Endogenous pannexin1 channels form functional intercellular cell-cell channels with characteristic voltage-dependent properties. Proc. Natl. Acad. Sci. USA.

[14] Demura K., Kusakizako T., Shihoya W., Hiraizumi M., Nomura K., Shimada H., Yamashita K., Nishizawa T., Taruno A., Nureki O. (2020). Cryo-EM structures of calcium homeostasis modulator channels in diverse oligomeric assemblies. Sci. Adv..

[15] Drożdżyk K., Sawicka M., Bahamonde-Santos M.I., Jonas Z., Deneka D., Albrecht C., Dutzler R. (2020). Cryo-EM structures and functional properties of CALHM channels of the human placenta. Elife.

[16] Ren Y., Li Y., Wang Y., Wen T., Lu X., Chang S., Zhang X., Shen Y., Yang X. (2022). Cryo-EM structure of the heptameric calcium homeostasis modulator 1 channel. J. Biol. Chem..

[17] Danielli S., Ma Z., Pantazi E., Kumar A., Demarco B., Fischer F.A., Paudel U., Weissenrieder J., Lee R.J., Joyce S. (2023). The ion channel CALHM6 controls bacterial infection-induced cellular cross-talk at the immunological synapse. EMBO J..

[18] Drozdzyk K., Peter M., Dutzler R. (2024). Structural features of heteromeric channels composed of CALHM2 and CALHM4 paralogs. Elife.

[19] Polfer R., Furukawa H. (2025). Biology, function and structure of the calcium homeostasis modulator family. J. Physiol..

[20] Ma Z., Paudel U., Wang M., Foskett J.K. (2025). A mechanism of CALHM1 ion channel gating. Am. J. Physiol. Cell Physiol..

[21] Taujale R., Park S.J., Gravel N., Soleymani S., Carter R., Boyd K., Keuning S.I., Ruan Z., Lu W., Kannan N. (2026). Identification and classification of ion channels across the tree of life provide functional insights into understudied CALHM channels. Elife.

[22] Deneka D., Sawicka M., Lam A.K.M., Paulino C., Dutzler R. (2018). Structure of a volume-regulated anion channel of the LRRC8 family. Nature.

[23] Kasuya G., Nakane T., Yokoyama T., Jia Y., Inoue M., Watanabe K., Nakamura R., Nishizawa T., Kusakizako T., Tsutsumi A. (2018). Cryo-EM structures of the human volume-regulated anion channel LRRC8. Nat. Struct. Mol. Biol..

[24] Kefauver J.M., Saotome K., Dubin A.E., Pallesen J., Cottrell C.A., Cahalan S.M., Qiu Z., Hong G., Crowley C.S., Whitwam T. (2018). Structure of the human volume regulated anion channel. Elife.

[25] Kern D.M., Bleier J., Mukherjee S., Hill J.M., Kossiakoff A.A., Isacoff E.Y., Brohawn S.G. (2023). Structural basis for assembly and lipid-mediated gating of LRRC8A:C volume-regulated anion channels. Nat. Struct. Mol. Biol..

[26] Nakamura R., Numata T., Kasuya G., Yokoyama T., Nishizawa T., Kusakizako T., Kato T., Hagino T., Dohmae N., Inoue M. (2020). Cryo-EM structure of the volume-regulated anion channel LRRC8D isoform identifies features important for substrate permeation. Commun. Biol..

[27] Qiu Z., Dubin A.E., Mathur J., Tu B., Reddy K., Miraglia L.J., Reinhardt J., Orth A.P., Patapoutian A. (2014). SWELL1, a plasma membrane protein, is an essential component of volume-regulated anion channel. Cell.

[28] Takahashi H., Yamada T., Denton J.S., Strange K., Karakas E. (2023). Cryo-EM structures of an LRRC8 chimera with native functional properties reveal heptameric assembly. Elife.

[29] Voss F.K., Ullrich F., Munch J., Lazarow K., Lutter D., Mah N., Andrade-Navarro M.A., von Kries J.P., Stauber T., Jentsch T.J. (2014). Identification of LRRC8 heteromers as an essential component of the volume-regulated anion channel VRAC. Science.

[30] Karakas E., Strange K., Denton J.S. (2025). Recent advances in structural characterization of volume-regulated anion channels (VRACs). J. Physiol..

[31] Cronier L., Defamie N., Dupays L., Théveniau-Ruissy M., Goffin F., Pointis G., Malassiné A. (2002). Connexin expression and gap junctional intercellular communication in human first trimester trophoblast. Mol. Hum. Reprod..

[32] Kibschull M., Gellhaus A., Carette D., Segretain D., Pointis G., Gilleron J. (2015). Physiological roles of connexins and pannexins in reproductive organs. Cell. Mol. Life Sci..

[33] Winterhager E., Kidder G.M. (2015). Gap junction connexins in female reproductive organs: Implications for women’s reproductive health. Hum. Reprod. Update.

[34] Xiao X., Tang Y., Wooff Y., Su C., Kang M., O’Carroll S.J., Chen Q., Chamley L. (2020). Upregulation of pannexin-1 hemichannels explains the apparent death of the syncytiotrophoblast during human placental explant culture. Placenta.

[35] Kubota K., Kim J.Y., Sawada A., Tokimasa S., Fujisaki H., Matsuda-Hashii Y., Ozono K., Hara J. (2004). LRRC8 involved in B cell development belongs to a novel family of leucine-rich repeat proteins. FEBS Lett..

[36] Keenen M.M., Jalihal A., Gladfelter A.S. (2025). Achieving multifunctionality in a single, tissue-sized syncytiotrophoblast cell in humans. Placenta.

[37] Frendo J.L., Cronier L., Bertin G., Guibourdenche J., Vidaud M., Evain-Brion D., Malassine A. (2003). Involvement of connexin 43 in human trophoblast cell fusion and differentiation. J. Cell Sci..

[38] Malassiné A., Cronier L. (2005). Involvement of gap junctions in placental functions and development. Biochim. Biophys. Acta.

[39] Otto T., Gellhaus A., Luschen N., Scheidler J., Bendix I., Dunk C., Wolf N., Lennartz K., Koninger A., Schmidt M. (2015). Oxygen Sensitivity of Placental Trophoblast Connexins 43 and 46: A Role in Preeclampsia?. J. Cell. Biochem..

[40] Chang C.W., Wakeland A.K., Parast M.M. (2018). Trophoblast lineage specification, differentiation and their regulation by oxygen tension. J. Endocrinol..

[41] Nishimura T., Dunk C., Lu Y., Feng X., Gellhaus A., Winterhager E., Rossant J., Lye S.J. (2004). Gap junctions are required for trophoblast proliferation in early human placental development. Placenta.

[42] Tan J.Y., Yeoh H.X.Y., Chia W.K., Tan J.W., Aizuddin A.N., Farouk W.I., Alfian N., Wong Y.P., Tan G.C. (2024). Overexpression of Connexin 40 in the Vascular Endothelial Cells of Placenta with Acute Chorioamnionitis. Diagnostics.

[43] Brisset A.C., Isakson B.E., Kwak B.R. (2009). Connexins in vascular physiology and pathology. Antioxid. Redox Signal..

[44] Figueroa X.F., Duling B.R. (2009). Gap junctions in the control of vascular function. Antioxid. Redox Signal..

[45] Pogoda K., Kameritsch P., Mannell H., Pohl U. (2019). Connexins in the control of vasomotor function. Acta Physiol..

[46] Pohl U. (2020). Connexins: Key Players in the Control of Vascular Plasticity and Function. Physiol. Rev..

[47] Yang Y., Delalio L.J., Best A.K., Macal E., Milstein J., Donnelly I., Miller A.M., McBride M., Shu X., Koval M. (2020). Endothelial Pannexin 1 Channels Control Inflammation by Regulating Intracellular Calcium. J. Immunol..

[48] Filiberto A.C., Spinosa M.D., Elder C.T., Su G., Leroy V., Ladd Z., Lu G., Mehaffey J.H., Salmon M.D., Hawkins R.B. (2022). Endothelial pannexin-1 channels modulate macrophage and smooth muscle cell activation in abdominal aortic aneurysm formation. Nat. Commun..

[49] Tachikawa M., Murakami K., Akaogi R., Akanuma S.I., Terasaki T., Hosoya K.I. (2020). Polarized hemichannel opening of pannexin 1/connexin 43 contributes to dysregulation of transport function in blood-brain barrier endothelial cells. Neurochem. Int..

[50] Morley L.C., Shi J., Gaunt H.J., Hyman A.J., Webster P.J., Williams C., Forbes K., Walker J.J., Simpson N.A.B., Beech D.J. (2018). Piezo1 channels are mechanosensors in human fetoplacental endothelial cells. Mol. Hum. Reprod..

[51] Wang S., Chennupati R., Kaur H., Iring A., Wettschureck N., Offermanns S. (2016). Endothelial cation channel PIEZO1 controls blood pressure by mediating flow-induced ATP release. J. Clin. Investig..

[52] López X., Palacios-Prado N., Guiza J., Escamilla R., Fernández P., Vega J.L., Rojas M., Marquez-Miranda V., Chamorro E., Cárdenas A.M. (2021). A physiologic rise in cytoplasmic calcium ion signal increases pannexin1 channel activity via a C-terminus phosphorylation by CaMKII. Proc. Natl. Acad. Sci. USA.

[53] Winterhager E., Grümmer R., Jahn E., Willecke K., Traub O. (1993). Spatial and temporal expression of connexin26 and connexin43 in rat endometrium during trophoblast invasion. Dev. Biol..

[54] Grümmer R., Chwalisz K., Mulholland J., Traub O., Winterhager E. (1994). Regulation of connexin26 and connexin43 expression in rat endometrium by ovarian steroid hormones. Biol. Reprod..

[55] Jahn E., Classen-Linke I., Kusche M., Beier H.M., Traub O., Grümmer R., Winterhager E. (1995). Expression of gap junction connexins in the human endometrium throughout the menstrual cycle. Hum. Reprod..

[56] Yu J., Berga S.L., Johnston-MacAnanny E.B., Sidell N., Bagchi I.C., Bagchi M.K., Taylor R.N. (2016). Endometrial Stromal Decidualization Responds Reversibly to Hormone Stimulation and Withdrawal. Endocrinology.

[57] Laws M.J., Taylor R.N., Sidell N., DeMayo F.J., Lydon J.P., Gutstein D.E., Bagchi M.K., Bagchi I.C. (2008). Gap junction communication between uterine stromal cells plays a critical role in pregnancy-associated neovascularization and embryo survival. Development.

[58] Yu J., Wu J., Bagchi I.C., Bagchi M.K., Sidell N., Taylor R.N. (2011). Disruption of gap junctions reduces biomarkers of decidualization and angiogenesis and increases inflammatory mediators in human endometrial stromal cell cultures. Mol. Cell. Endocrinol..

[59] Tittarelli A., Mendoza-Naranjo A., Farias M., Guerrero I., Ihara F., Wennerberg E., Riquelme S., Gleisner A., Kalergis A., Lundqvist A. (2014). Gap junction intercellular communications regulate NK cell activation and modulate NK cytotoxic capacity. J. Immunol..

[60] Ajasin D., Velasquez S., Gibson J., Scemes E., Cibelli A., Spray D., Eugenin E.A. (2025). Pannexin-1 channels, extracellular ATP, and purinergic receptors are essential for CCR5/CXCR4 clustering and HIV entry. NeuroImmune Pharm. Ther..

[61] Chen W., Zhu S., Wang Y., Li J., Qiang X., Zhao X., Yang H., D’Angelo J., Becker L., Wang P. (2019). Enhanced Macrophage Pannexin 1 Expression and Hemichannel Activation Exacerbates Lethal Experimental Sepsis. Sci. Rep..

[62] Eugenín E.A., Brañes M.C., Berman J.W., Sáez J.C. (2003). TNF-alpha plus IFN-gamma induce connexin43 expression and formation of gap junctions between human monocytes/macrophages that enhance physiological responses. J. Immunol..

[63] Sagrillo-Fagundes L., Casagrande Paim T., Pretto L., Bertaco I., Zanatelli C., Vaillancourt C., Wink M.R. (2022). The implications of the purinergic signaling throughout pregnancy. J. Cell. Physiol..

[64] Szabo D., Tod P., Goloncser F., Roman V., Lendvai B., Otrokocsi L., Sperlagh B. (2022). Maternal P2X7 receptor inhibition prevents autism-like phenotype in male mouse offspring through the NLRP3-IL-1beta pathway. Brain Behav. Immun..

[65] Elgueta R., Tobar J.A., Shoji K.F., De Calisto J., Kalergis A.M., Bono M.R., Rosemblatt M., Sáez J.C. (2009). Gap junctions at the dendritic cell-T cell interface are key elements for antigen-dependent T cell activation. J. Immunol..

[66] Chen K.W., Demarco B., Broz P. (2020). Pannexin-1 promotes NLRP3 activation during apoptosis but is dispensable for canonical or noncanonical inflammasome activation. Eur. J. Immunol..

[67] Liu C., Shen Y., Huang L., Wang J. (2022). TLR2/caspase-5/Panx1 pathway mediates necrosis-induced NLRP3 inflammasome activation in macrophages during acute kidney injury. Cell Death Discov..

[68] Huang Y., Mao Z., Zhang Z., Obata F., Yang X., Zhang X., Huang Y., Mitsui T., Fan J., Takeda M. (2019). Connexin43 Contributes to Inflammasome Activation and Lipopolysaccharide-Initiated Acute Renal Injury via Modulation of Intracellular Oxidative Status. Antioxid. Redox Signal..

[69] Erlebacher A. (2013). Immunology of the maternal-fetal interface. Annu. Rev. Immunol..

[70] Moffett A., Colucci F. (2014). Uterine NK cells: Active regulators at the maternal-fetal interface. J. Clin. Investig..

[71] Sáez J.C., Berthoud V.M., Brañes M.C., Martínez A.D., Beyer E.C. (2003). Plasma membrane channels formed by connexins: Their regulation and functions. Physiol. Rev..

[72] Dukic A.R., Gerbaud P., Guibourdenche J., Thiede B., Taskén K., Pidoux G. (2018). Ezrin-anchored PKA phosphorylates serine 369 and 373 on connexin 43 to enhance gap junction assembly, communication, and cell fusion. Biochem. J..

[73] Pidoux G., Gerbaud P., Dompierre J., Lygren B., Solstad T., Evain-Brion D., Taskén K. (2014). A PKA-ezrin-Cx43 signaling complex controls gap junction communication and thereby trophoblast cell fusion. J. Cell Sci..

[74] Araya R., Eckardt D., Riquelme M.A., Willecke K., Sáez J.C. (2003). Presence and importance of connexin43 during myogenesis. Cell Commun. Adhes..

[75] Proulx A., Merrifield P.A., Naus C.C. (1997). Blocking gap junctional intercellular communication in myoblasts inhibits myogenin and MRF4 expression. Dev. Genet..

[76] Herde K., Hartmann S., Brehm R., Kilian O., Heiss C., Hild A., Alt V., Bergmann M., Schnettler R., Wenisch S. (2007). Connexin 43 expression of foreign body giant cells after implantation of nanoparticulate hydroxyapatite. Biomaterials.

[77] Munoz M.F., Griffith T.N., Contreras J.E. (2021). Mechanisms of ATP release in pain: Role of pannexin and connexin channels. Purinergic Signal..

[78] Kaufman E.K., Swartz T.H. (2026). Pannexin-1-mediated ATP signaling as a driver of immune communication and chronic inflammation in HIV infection. Front. Immunol..

[79] Ma Z., Taruno A., Ohmoto M., Jyotaki M., Lim J.C., Miyazaki H., Niisato N., Marunaka Y., Lee R.J., Hoff H. (2018). CALHM3 Is Essential for Rapid Ion Channel-Mediated Purinergic Neurotransmission of GPCR-Mediated Tastes. Neuron.

[80] Hyzinski-Garcia M.C., Rudkouskaya A., Mongin A.A. (2014). LRRC8A protein is indispensable for swelling-activated and ATP-induced release of excitatory amino acids in rat astrocytes. J. Physiol..

[81] Paz-Lopez S. (2025). ATP release mediated by pannexin-3 is required for plasma cell survival via P2X4 receptors in bone marrow. Purinergic Signal..

[82] Lovatt A., Butler J., Dale N. (2025). Mechanisms of permselectivity of connexin hemichannels to small molecules. J. Biol. Chem..

[83] Retamal M.A., Cortés C.J., Reuss L., Bennett M.V., Saez J.C. (2006). S-nitrosylation and permeation through connexin 43 hemichannels in astrocytes: Induction by oxidant stress and reversal by reducing agents. Proc. Natl. Acad. Sci. USA.

[84] Figueroa X.F., Lillo M.A., Gaete P.S., Riquelme M.A., Sáez J.C. (2013). Diffusion of nitric oxide across cell membranes of the vascular wall requires specific connexin-based channels. Neuropharmacology.

[85] Lillo M.A., Himelman E., Shirokova N., Xie L.H., Fraidenraich D., Contreras J.E. (2019). S-nitrosylation of connexin43 hemichannels elicits cardiac stress-induced arrhythmias in Duchenne muscular dystrophy mice. JCI Insight.

[86] Sáez J.C., Ocaranza F.J., Prieto-Villalobos J., Orellana J.A. (2025). Connexin and Pannexin Hemichannels: Broad-Spectrum Players in Neuroinflammatory Signaling. J. Neurochem..

[87] Taruno A., Vingtdeux V., Ohmoto M., Ma Z., Dvoryanchikov G., Li A., Adrien L., Zhao H., Leung S., Abernethy M. (2013). CALHM1 ion channel mediates purinergic neurotransmission of sweet, bitter and umami tastes. Nature.

[88] Ma J., Qi X., Yang C., Pan R., Wang S., Wu J., Huang L., Chen H., Cheng J., Wu R. (2018). Calhm2 governs astrocytic ATP releasing in the development of depression-like behaviors. Mol. Psychiatry.

[89] Sato Y. (2020). Endovascular trophoblast and spiral artery remodeling. Mol. Cell. Endocrinol..

[90] Pollheimer J., Vondra S., Baltayeva J., Beristain A.G., Knofler M. (2018). Regulation of Placental Extravillous Trophoblasts by the Maternal Uterine Environment. Front. Immunol..

[91] Qiu Y., Chen M., Lin R., Chen X., He L., Yin H., Chen X. (2025). Crosstalk between decidual natural killer cells and extravillous trophoblasts at the maternal-fetal interface: Current status and future perspectives. Front. Immunol..

[92] Rozas-Villanueva F.M., Orellana V.P., Alarcon R., Maripillan J., Martinez A.D., Alfaro I.E., Retamal M.A. (2024). Cx40 Levels Regulate Hypoxia-Induced Changes in the Migration, Proliferation, and Formation of Gap Junction Plaques in an Extravillous Trophoblast Cell Model. Cells.

[93] Duval F., Dos Santos E., Moindjie H., Serazin V., Swierkowski-Blanchard N., Vialard F., Dieudonné M.N. (2017). Adiponectin limits differentiation and trophoblast invasion in human endometrial cells. J. Mol. Endocrinol..

[94] Falk L., Dang-Lawson M., Vega J.L., Pournia F., Choi K., Jang C., Naus C.C., Matsuuchi L. (2014). Mutations of Cx43 that affect B cell spreading in response to BCR signaling. Biol. Open.

[95] Matsuuchi L., Naus C.C. (2013). Gap junction proteins on the move: Connexins, the cytoskeleton and migration. Biochim. Biophys. Acta.

[96] Pidoux G., Gerbaud P., Gnidehou S., Grynberg M., Geneau G., Guibourdenche J., Carette D., Cronier L., Evain-Brion D., Malassiné A. (2010). ZO-1 is involved in trophoblastic cell differentiation in human placenta. Am. J. Physiol. Cell Physiol..

[97] Chappell L.C., Cluver C.A., Kingdom J., Tong S. (2021). Pre-eclampsia. Lancet.

[98] El-Khalik S.R.A., Ibrahim R.R., Ghafar M.T.A., Shatat D., El-Deeb O.S. (2022). Novel insights into the SLC7A11-mediated ferroptosis signaling pathways in preeclampsia patients: Identifying pannexin 1 and toll-like receptor 4 as innovative prospective diagnostic biomarkers. J. Assist. Reprod. Genet..

[99] Huang Y., Bai Z., Sui S. (2024). miR-224-5p alleviates preeclampsia-like mouse symptoms by targeting PANX1 to inhibit ferroptosis in trophoblast cells. Placenta.

[100] Tersigni C., Onori M., Beneduce G., Sannino F., Franco R., Busnelli A., Granieri C., Milardi D., Pontecorvi A., Lanzone A. (2025). Primary versus secondary recurrent pregnancy losses: Clinical findings and live birth rate after comprehensive work-up and personalized management. Acta Obstet. Gynecol. Scand..

[101] He X., Chen Q. (2016). Reduced expressions of connexin 43 and VEGF in the first-trimester tissues from women with recurrent pregnancy loss. Reprod. Biol. Endocrinol..

[102] Nair R.R., Jain M., Singh K. (2011). Reduced expression of gap junction gene connexin 43 in recurrent early pregnancy loss patients. Placenta.

[103] Resnik R. (2002). Intrauterine growth restriction. Obstet. Gynecol..

[104] Burton G.J., Jauniaux E. (2018). Pathophysiology of placental-derived fetal growth restriction. Am. J. Obstet. Gynecol..

[105] Flenniken A.M., Osborne L.R., Anderson N., Ciliberti N., Fleming C., Gittens J.E., Gong X.Q., Kelsey L.B., Lounsbury C., Moreno L. (2005). A Gja1 missense mutation in a mouse model of oculodentodigital dysplasia. Development.

[106] Winterhager E., Gellhaus A., Blois S.M., Hill L.A., Barr K.J., Kidder G.M. (2013). Decidual angiogenesis and placental orientation are altered in mice heterozygous for a dominant loss-of-function Gja1 (connexin43) mutation. Biol. Reprod..

[107] Creswell L., Rolnik D.L., Lindow S.W., O’Gorman N. (2023). Preterm Birth: Screening and Prediction. Int. J. Womens Health.

[108] Jung E., Romero R., Suksai M., Gotsch F., Chaemsaithong P., Erez O., Conde-Agudelo A., Gomez-Lopez N., Berry S.M., Meyyazhagan A. (2024). Clinical chorioamnionitis at term: Definition, pathogenesis, microbiology, diagnosis, and treatment. Am. J. Obstet. Gynecol..

[109] Costa E., Okesola B.O., Thrasivoulou C., Becker D.L., Deprest J.A., David A.L., Chowdhury T.T. (2021). Cx43 mediates changes in myofibroblast contraction and collagen release in human amniotic membrane defects after trauma. Sci. Rep..

[110] Costa E., Thrasivoulou C., Becker D.L., Deprest J.A., David A.L., Chowdhury T.T. (2023). Cx43 regulates mechanotransduction mechanisms in human preterm amniotic membrane defects. Prenat. Diagn..

[111] Costa E., Thrasivoulou C., Becker D.L., Deprest J., David A.L., Chowdhury T.T. (2025). Role of Myofibroblasts in the Repair of Iatrogenic Preterm Membranes Subjected to Mechanical Stimulation. Prenat. Diagn..

[112] Miyoshi H., Konishi H., Teraoka Y., Urabe S., Furusho H., Miyauchi M., Takata T., Kudo Y. (2016). Enhanced Expression of Contractile-Associated Proteins and Ion Channels in Preterm Delivery Model Mice with Chronic Odontogenic Porphyromonas Gingivalis Infection. Reprod. Sci..

[113] Yu J., Boicea A., Barrett K.L., James C.O., Bagchi I.C., Bagchi M.K., Nezhat C., Sidell N., Taylor R.N. (2014). Reduced connexin 43 in eutopic endometrium and cultured endometrial stromal cells from subjects with endometriosis. Mol. Hum. Reprod..

[114] Nevin R.L. (2012). Mefloquine gap junction blockade and risk of pregnancy loss. Biol. Reprod..

[115] Boeldt D.S., Hankes A.C., Alvarez R.E., Khurshid N., Balistreri M., Grummer M.A., Yi F., Bird I.M. (2014). Pregnancy programming and preeclampsia: Identifying a human endothelial model to study pregnancy-adapted endothelial function and endothelial adaptive failure in preeclamptic subjects. Adv. Exp. Med. Biol..

[116] Knize M., Pitha J., Hubacek J.A., Fait T. (2021). The role of connexin 37 polymorphism in spontaneous abortion. Physiol. Res..

[117] Sang Q., Zhang Z., Shi J., Sun X., Li B., Yan Z., Xue S., Ai A., Lyu Q., Li W. (2019). A pannexin 1 channelopathy causes human oocyte death. Sci. Transl. Med..

[118] Wang W., Qu R., Dou Q., Wu F., Wang W., Chen B., Mu J., Zhang Z., Zhao L., Zhou Z. (2021). Homozygous variants in PANX1 cause human oocyte death and female infertility. Eur. J. Hum. Genet..

[119] Zhou J., Mao R., Wang M., Long R., Gao L., Wang X., Jin L., Zhu L. (2024). A novel heterozygous missense variant of PANX1 causes human oocyte death and female infertility. J. Ovarian Res..

[120] Bruzzone R., Barbe M.T., Jakob N.J., Monyer H. (2005). Pharmacological properties of homomeric and heteromeric pannexin hemichannels expressed in Xenopus oocytes. J. Neurochem..

[121] Spray D.C., Ye Z.C., Ransom B.R. (2006). Functional connexin “hemichannels”: A critical appraisal. Glia.

[122] Vessey J.P., Lalonde M.R., Mizan H.A., Welch N.C., Kelly M.E., Barnes S. (2004). Carbenoxolone inhibition of voltage-gated Ca channels and synaptic transmission in the retina. J. Neurophysiol..

[123] Jellinck P.H., Monder C., McEwen B.S., Sakai R.R. (1993). Differential inhibition of 11 beta-hydroxysteroid dehydrogenase by carbenoxolone in rat brain regions and peripheral tissues. J. Steroid Biochem. Mol. Biol..

[124] Vo D.K., Nguyen T.T., Joo S.A., Maeng H.J. (2026). Calcitriol-mediated modulation of organic anion transporters: Insights from endogenous biomarkers and methotrexate pharmacokinetics. Eur. J. Pharm. Sci..

[125] Rocereta J.A., Sturhahn T., Pumroy R.A., Fricke T.C., Herzog C., Leffler A., Moiseenkova-Bell V. (2025). Structural insights into TRPV2 modulation by probenecid. Nat. Struct. Mol. Biol..

[126] King D.R., Sedovy M.W., Leng X., Xue J., Lamouille S., Koval M., Isakson B.E., Johnstone S.R. (2021). Mechanisms of Connexin Regulating Peptides. Int. J. Mol. Sci..

[127] Lagos C.F., Vargas A., García A., Duarte Y., Yi C., Sáez J.C. (2026). Structure-based virtual screening for selective connexin hemichannel blockers: A historical perspective on the discovery of a small organic inhibitor. Front. Pharmacol..

[128] Kaneko Y., Tachikawa M., Akaogi R., Fujimoto K., Ishibashi M., Uchida Y., Couraud P.O., Ohtsuki S., Hosoya K., Terasaki T. (2015). Contribution of pannexin 1 and connexin 43 hemichannels to extracellular calcium-dependent transport dynamics in human blood-brain barrier endothelial cells. J. Pharmacol. Exp. Ther..

[129] Santiquet N., Robert C., Richard F.J. (2013). The dynamics of connexin expression, degradation and localisation are regulated by gonadotropins during the early stages of in vitro maturation of swine oocytes. PLoS ONE.

[130] Perez-Armendariz E.M., Luna J., Miranda C., Talavera D., Romano M.C. (1996). Luteinizing and human chorionic gonadotropin hormones increase intercellular communication and gap junctions in cultured mouse leydig cells. Endocrine.

[131] Maertens C., Droogmans G., Chakraborty P., Nilius B. (2001). Inhibition of volume-regulated anion channels in cultured endothelial cells by the anti-oestrogens clomiphene and nafoxidine. Br. J. Pharmacol..

[132] Arutyunyan A., Roberts K., Troule K., Wong F.C.K., Sheridan M.A., Kats I., Garcia-Alonso L., Velten B., Hoo R., Ruiz-Morales E.R. (2023). Spatial multiomics map of trophoblast development in early pregnancy. Nature.

[133] Karvas R.M., Khan S.A., Verma S., Yin Y., Kulkarni D., Dong C., Park K.M., Chew B., Sane E., Fischer L.A. (2022). Stem-cell-derived trophoblast organoids model human placental development and susceptibility to emerging pathogens. Cell Stem Cell.

[134] Shannon M.J., McNeill G.L., Koksal B., Baltayeva J., Wachter J., Castellana B., Penaherrera M.S., Robinson W.P., Leung P.C.K., Beristain A.G. (2024). Single-cell assessment of primary and stem cell-derived human trophoblast organoids as placenta-modeling platforms. Dev. Cell.

[135] Li Q., Cui C., Liao R., Yin X., Wang D., Cheng Y., Huang B., Wang L., Yan M., Zhou J. (2023). The pathogenesis of common Gjb2 mutations associated with human hereditary deafness in mice. Cell. Mol. Life Sci..

[136] Kannan A., Beal J.R., Neff A.M., Bagchi M.K., Bagchi I.C. (2023). Runx1 regulates critical factors that control uterine angiogenesis and trophoblast differentiation during placental development. Proc. Natl. Acad. Sci. USA.

[137] Peng Q., Yue C., Chen A.C.H., Lee K.C., Fong S.W., Yeung W.S.B., Lee Y.L. (2019). Connexin 43 is involved in early differentiation of human embryonic stem cells. Differentiation.

[138] Yu J., Berga S.L., Zou W., Yook D.G., Pan J.C., Andrade A.A., Zhao L., Sidell N., Bagchi I.C., Bagchi M.K. (2017). IL-1beta Inhibits Connexin 43 and Disrupts Decidualization of Human Endometrial Stromal Cells Through ERK1/2 and p38 MAP Kinase. Endocrinology.

[139] Kibschull M., Lye S.J., Shynlova O. (2016). Generation and Use of Trophoblast Stem Cells and Uterine Myocytes to Study the Role of Connexins for Pregnancy and Labor. Methods Mol. Biol..

