# Supplementary material for: Large-Pore Channels at the Maternal–Fetal Interface: Progress and Open Research Avenues
Source: Biology (Basel). 2026 Sep 8;15(18):1571. doi: 10.3390/biology15181571 (PMC13603352; doi:10.3390/biology15181571)
Supplement: Supplementary file 1 [file biology-15-01571-s001.zip › biology-4509033-supplementary.pdf]

Table S1. A decade (2015–2025) of progress in placental connexin research.

Year	Isoform	Key Finding	Reference
**2025**	Cx43	Cx43 plaque formation at iatrogenic amniotic membrane defects hinders repair; antisense treatment restores collagen and elastin levels.	[111]
**2024**	Cx40	Cx40 expression modulates migration, proliferation, and the assembly of gap junction plaques in an EVT cell model.	[92]
**2024**	Cx40	Cx40 overexpression in maternal and fetal placental vasculature during chorioamnionitis.	[42]
**2023**	Cx43	Mechanical stimulation and trauma upregulate Cx43 expression in preterm amniotic membrane defects; Cx43 inhibition reduces PGE_2 levels.	[110]
**2023**	Cx26	Generation of *Gjb2*^{35delG}/^{35delG} homozygous mutant mice demonstrated that Cx26 is indispensable for placental development and embryonic viability.	[135]
**2023**	Cx43	The loss of RUNX1 disrupts *GJA1*-mediated angiogenesis and impairs trophoblast differentiation and spiral artery remodeling.	[136]

 https://doi.org/10.3390/biology15181571

**2019**	Cx43	Cx43 knockdown in human embryonic stem cells enhances differentiation into trophoblast-like cells, increasing human chorionic gonadotropin secretion, cell invasion, and the expression of implantation-related genes.	[137]
**2018**	Cx43	PKA-mediated phosphorylation of Cx43 at Serine 369 and Serine 373 triggers gap junction assembly and communication, which are essential for human cytotrophoblast fusion and syncytialization.	[72]
**2017**	Cx43	IL-1β-induced inflammation downregulates Cx43 in human endometrial stromal cells via ERK1/2 and p38 MAPK pathways, impairing endometrial receptivity.	[138]
**2017**	Cx43	Adiponectin acts through ADIPOR1/2 to downregulate Cx43 in endometrial stromal cells, exerting an anti-differentiative effect that restricts trophoblast invasion via modulated MMP/TIMP activity.	[93]
**2016**	Cx43	Downregulation of Cx43 in first-trimester chorionic villi and decidua of women with recurrent pregnancy loss.	[101]
**2016**	Cx31, Cx31.1, Cx26, Cx43	Established *in vitro* models using Cx-deficient trophoblast stem cells and primary myocytes to study trophoblast differentiation and mechanotransduction in myometrial growth.	[139]

https://doi.org/10.3390/biology15181571

**2016**	Cx43	Cx43 expression is reversibly regulated by ovarian hormones and cAMP, acting as a master driver of mesenchymal-epithelial transition in human endometrial stromal cells.	[56]
**2016**	Cx43	Chronic systemic inflammation induced by *Porphyromonas gingivalis* infection triggers premature upregulation of myometrial Cx43, leading to early onset of labor and preterm delivery.	[112]
**2015**	Cx43, Cx46	Acute hypoxia increases Cx43, while chronic hypoxia induces HIF-2α-dependent Cx43 internalization and Cx46 downregulation.	[39]

Note: For a comprehensive summary of studies and findings prior to 2015, please refer to the reviews [32,33].

Table S2. Evidence classification justification for key claims.

Claim	Limitations	Justification	Species	References
Cx43 required for syncytialization	siRNA in BeWo cells; limited ultrastructural evidence	Multiple independent studies with complementary approaches	Human	[31,37]
Panx1 upregulation in explants	Probenecid non-specific; no genetic validation	Strong evidence but requires follow-up	Human	[34]
CALHM2/4/6 in trophoblasts	Transcript detected; protein expression and functional activity not confirmed	Reproducible but awaiting independent replication	Human	[15]
LRRC8 transcripts in placenta	Incidental finding; no cell-type resolution	Hypothesis-generating only	Human	[35]
Cx40 regulates EVT proliferation or invasion	JEG-3 cell line; requires primary cell validation	Promising but not yet established in primary cells	Human	[41,92]
Panx1–ferroptosis in preeclampsia	Small cohorts; mouse model limitations	Compelling but preliminary	Human	[98,99]

https://doi.org/10.3390/biology15181571
